# Editorial: The use of microbial volatile compounds for controlling plant pathogens

**DOI:** 10.3389/fpls.2024.1502485

**Published:** 2024-10-17

**Authors:** Elsherbiny A. Elsherbiny, Alessandra Di Francesco, Joan W. Bennett

**Affiliations:** ^1^ Plant Pathology Department, Faculty of Agriculture, Mansoura University, Mansoura, Egypt; ^2^ Department of Agriculture, Food, Environmental and Animal Sciences, University of Udine, Udine, Italy; ^3^ Department of Plant Biology, Rutgers, The State University of New Jersey, New Brunswick, NJ, United States

**Keywords:** volatile compounds, secondary metabolites, phytopathogens, plant defense, biocontrol

There is growing evidence that microbial volatile organic compounds (VOCs) regulate many plant and fungal developmental processes, mediate signals that lead to mutualistic relationships such as mycorrhizal formation, and – the subject of this Research Topic - act as effective antimicrobial agents against various plant pathogens.

VOCs are low molecular weight, usually lipophilic metabolites that easily evaporate at room temperature. Most VOCs have characteristic odors, so it is not surprising that a great deal of historical volatile research has been conducted by either food and flavor researchers or perfume chemists. Current microbial metabolomic studies predominantly focus on nonvolatile, often water-soluble metabolites, and overlook VOCs. However, in terrestrial environments, water is not constantly present. Many inter-organism interactions are mediated by VOCs that can travel through water and air. Volatile-facilitated intercellular communication processes are common in nature but understudied in the laboratory.

The total profile of volatiles emitted by an organism at a specific sampling time can be described as its ‘volatilome’, and it is common for the same VOC to be produced by dozens, if not hundreds, of species. Approximately 2000 VOCs have been documented from microbial sources of which 300 have been identified from fungi. More than 1700 VOCs have been found from plants. Since the number of known microbial species is thought to be poorly sampled, there is likely a great deal of untapped VOC diversity yet to be discovered.

In recent years, agriculturalists and other applied biologists have come to appreciate that VOCs may be important components of the biocontrol process. This Research Topic “The Use of Microbial Volatile Compounds for Controlling Plant Pathogens” demonstrates the myriad ways that VOCs can serve as biofumigants and thereby offer promising alternatives to chemical fungicides and bactericides. Furthermore, this Research Topic speaks to the breadth, depth, and diversity of biological activities mediated by these small gas-phase molecules. Di Francesco et al. demonstrate that 3-methyl-1-butanol (=isopentyl alcohol) and 1-nonene, components of the volatilome of *Pseudomonas synxantha*, served as biofumigants against kiwifruit pathogens. Elizarraraz-Martínez et al. report on the impact of nonanal treatment on common bean plant yields, focusing on the phenolic compounds and antioxidant content, as well as the physical characteristics of the beans.

Alvarez-Garcia et al. used essential oils of oregano, thyme, lavender, and other herbs to inhibit the growth of species of two major post-harvest fungal pathogens, namely gray mold (*Botrytis* sp.) and brown rot (*Monilinia* sp.). Essential oils with higher levels of carvacrol and thymol showed the highest antifungal activity. Adra et al. sought volatile biocontrol agents from the inside of termite guts and found VOC-emitting *Streptomyces* strains that not only inhibited the growth of *Pyrrhoderma moxium* (causes extensive damage to many fruit, ornamental, herbaceous, and coniferous trees in different regions) but also yielded VOCs that enhanced root, shoot and chlorophyll production in *Arabidopsis thaliana*. Kim et al. found that VOCs emitted by two species of *Trichoderma* were effective in inhibiting the mycelial growth of red pepper anthracnose caused by *Colletotrichum acutatum*. Mendoza-Mendoza et al. review the abiotic and biotic interactions that regulate the synthesis of 6-pentyl-alpha-pyrone (6-PP), a ketone generated by Trichoderma fungi, and its impact on plant pathogens through both direct and indirect mechanisms such as induced systemic resistance.

Finally, the work by Wu et al. is notable in that it focused on the mechanism of action displayed by 2-heptanol (= heptan-2-ol) in suppressing the growth of *Botrytis cinerea*, the necrotrophic gray mold fungus that affects grapes, tomatoes, and other high-value plant crops. Because so little is known about the molecular mechanism of VOC action, it is an important finding that 2-heptanol disrupted membrane transport and increased amino acid transport, ultimately yielding nutrient depletion and cell death.

In summary, we hope that this Research Topic will inspire more scientists to recognize that the chemical accomplishments of fungi and streptomycetes do not end with famous pharmaceutically active natural products like penicillin and streptomycin, but extend to small, gas-phase molecules that pathogens can ‘breathe in’. The semiotic organic chemical signals that fungi and other microbes use when communicating in the gas phase is a language waiting to be deciphered and then rationally applied for plant pathogen control.

